# Practices of dietitians in dialysis units in Brazil: nutritional assessment and intervention

**DOI:** 10.1590/2175-8239-JBN-2024-0112en

**Published:** 2024-09-06

**Authors:** Viviane de Oliveira Leal, Denise Mafra

**Affiliations:** 1 Universidade do Estado do Rio de Janeiro, Hospital Universitário Pedro Ernesto, Divisão de Nutrição, Rio de Janeiro, RJ, Brazil.; 2 Centro de Nefrologia Mageense, Magé, RJ, Brazil.; 3 Universidade Federal Fluminense, Programa de Pós-Graduação em Ciências Médicas, Niterói, RJ, Brazil.; 4 Universidade Federal do Rio de Janeiro, Programa de Pós-Graduação em Ciências Biológicas, Fisiologia, Rio de Janeiro, RJ, Brazil.

Nutrition plays a crucial role in preventing and controlling metabolic and nutritional disorders in patients with chronic kidney disease (CKD) undergoing dialysis. However, as reported in the article by Nerbass *et al*.^1^, “Dietitians’ practices in dialysis units in Brazil: nutritional assessment and intervention,” the use of tools to assess nutritional status, as well as intervention strategies in cases of nutritional risk/malnutrition, remains heterogeneous.

In 2020, the National Kidney Foundation, in the Kidney Disease Outcomes Quality Initiative (NKF/KDOQI) document, suggested specific guidelines for the nutritional monitoring of CKD patients^2^. Therefore, this guideline may be considered the fundamental instrument to guide practices related to nutritional diagnosis and support nutritional approaches for patients undergoing dialysis.

Nutritional risk screening, commonly used in hospital settings, is considered an initial, quick, and easy step to identify patients at nutritional risk early. In addition to screening, nutritional assessment should be routinely performed at a dialysis clinic. Once a nutritional deficit has been identified, the patient should receive closer attention and treatment from the clinic team and, naturally, from the dietitian, who should provide appropriate interventions. Thus, routine nutritional assessment and data interpretation allow for identifying the causes and severity of nutrition-related disorders while guiding more assertive nutritional therapy^3^.

Therefore, although nutritional screening might be perceived as “just another” tool, it is essential to systematize the need for nutritional assessment. Another interesting point is that other health professionals, patients, or their support network could perform nutritional screening. According to the NKF/KDOQI (2020), nutritional risk screening should be conducted every six months, and since there is no indication of a preferred tool, the Malnutrition Screening Tool (MST) could be used. The MST incorporates data quickly obtained from individual dialysis records and patient anamnesis (such as recent weight loss and reduction in appetite).

The nutritional assessment, for its part, should include dietary intake, appetite, anthropometric data (body weight, skinfold thickness, body mass index), biochemical data, and physical examination^2^. Given the absence of a single, comprehensive indicator for the nutritional diagnosis of CKD patients undergoing dialysis, composite methods are essential tools in clinical practice.

The Subjective Global Assessment (SGA), which encompasses almost all the nuances of nutritional assessment recommended by the NKF/KDOQI (2020), is the nutrition assessment tool used by more than 80% of dietitians in Brazil^1^. Since it has been validated as a predictor of mortality in CKD patients, the 7-point SGA is especially recommended for assessing the nutrition status of dialysis patients^2^. Specifically for hemodialysis (HD) patients, the Malnutrition Inflammation Score (MIS) may also be used. Although it is more objective and comprehensive than the SGA, as it includes biochemical parameters (albumin and total iron-binding capacity) and body mass index, it is used by only 49% of dietitians^1^. However, it is worth noting that the nutritional diagnosis according to these tools (SGA and MIS) should ideally be complemented with additional anthropometric, biochemical, and dietary intake parameters^4^.

The frequency of nutritional assessment is also suggested by the NKF/KDOQI (2020)^2^: at dialysis initiation (within the first 90 days of treatment); when indicated by nutritional screening; annually; or based on clinical judgment. In the study conducted by Nerbass *et al*.^1^, the SGA was the tool with the highest usage percentage with a defined periodicity (53% in total, 36% every three to six months, 9% annually, and 8% monthly) This is likely due to its ease of execution and interpretation.

The workload of the dietitian is a crucial variable in determining the systematization of patient care (tools used and their frequency). Nevertheless, the difference in the varied application of tools for assessing nutritional status, irrespective of their periodicity, is small when dietitians are divided according to the number of patients seen per monthly working hour (using a cutoff value of 1.6). It is, therefore, challenging to state whether it would be more appropriate to use fewer tools at shorter intervals or more tools at more extended application periods.

However, it is a fact that 1.6 patients per working hour of the dietitian is significantly above the recommended by the Federal Council of Nutritionists (*Conselho Federal de Nutricionistas*, CFN) in Brazil, which establishes one dietitian for every 50 patients with a monthly workload of 30 hours per week (CFN, 2018)^5^. This would correspond to the ideal of 0.3 patients per working hour. Therefore, each dialysis unit must establish its priorities and capabilities within its technical routine, considering each service’s time management and available resources. The aim is to prioritize not only the diagnosis but also the continuity of nutritional care.

Nutritional intervention in cases of malnutrition, observed in the study by Nerbass *et al*.^1^, is in line with the recommendations of international guidelines, which determine the nutritional counseling as an initial intervention strategy^2^. Thus, increased food fractionation and portioning, including high-energy density and high-protein foods, is indicated. To monitor adherence and provide relevant adjustments, increasing the frequency of attendance is necessary and is indeed described by most dietitians.

When there is still a gap between actual intake and the target needs/objectives of nutritional treatment, oral nutritional supplementation (ONS) should be considered. For this, a few details are important^2,6^:

- Follow-up period for ONS should be at least three months;
- ONS should be prescribed 2 to 3 times a day. To avoid compromising regular food intake (due to greater satiety), supplementation should be administered 1 hour after the main meals. Another option would be to use it as the last meal of the day (bedtime snack), to mitigate overnight fasting, or even during the HD procedure to ease/reverse catabolism. In this case, potential complications (such as gastrointestinal symptoms and postprandial hypotension) should be managed with close monitoring;
- ONS prescription should be based on the patient’s preferences, considering different possible presentations (bars, powders, ready-to-drink preparations);
- In cases where there is no need for fluid and electrolyte restriction, standard composition formulas may be used, always with intensive monitoring.

These details are crucial when selecting the appropriate ONS, as they will require use for a considerable period, resulting in higher costs. Furthermore, the sensory characteristics of the chosen supplement/module should be pleasant enough to promote long-term adherence.

In Brazil, only 17% of dialysis units provide supplementation (either to all or just some of the patients with an indication). This percentage varies between regions of the country^1^. Thus, specific public policies for supplement donation or the feasibility of additional financial resources for dialysis patients diagnosed with malnutrition and in situations of social vulnerability could be articulated at the national level.

Since patients often bear the cost of ONS themselves, using nutrient modules (indicated by 63% of dietitians) or industrialized formulas should be considered based on individual clinical and biochemical needs and financial constraints. Surprisingly, the percentage of recommendations for both standard formulas and those specifically for CKD in dialysis was no different (around 78%) despite the considerably higher cost of specific formulas.

Although industrialized formulas designed for dialysis patients may seem safer, they are not free of potassium and phosphorus, requiring careful selection. Standard formulas also have highly variable electrolyte content. However, the availability of unflavored versions could make dietary interventions more versatile, as they may be used in different types of preparations (e.g., milk in smoothies and porridge, pancake batter, or savory preparations such as purees and soups).

Considering all these possibilities for assessing nutritional status and, especially, for assertive dietary interventions, the dietitian’s importance in monitoring dialysis patients becomes even more evident. Proper and individualized dietary care is a fundamental cornerstone of the treatment for CKD patients on dialysis.

Regarding the research conducted by Nerbass *et al*.^1^, it is worth highlighting the necessity of greater engagement from clinic dietitians in this study, as only 24% responded to the questionnaire. An electronic questionnaire shared on social media and messaging apps is appropriate for reaching many professionals. However, as the authors discuss, it may introduce a sampling bias.

Finally, the study addresses a relevant and necessary issue regarding nutritional practices in dialysis units in Brazil, providing material for future studies to explore the variability of these practices further and conduct more detailed regional analyses.
