# Supplementary material for: Practices of dietitians in dialysis units in Brazil: nutritional assessment and intervention
Source: J Bras Nefrol. 2024 Sep 6;46(3):e20240112. doi: 10.1590/2175-8239-JBN-2024-0112en (PMC11379405; doi:10.1590/2175-8239-JBN-2024-0112en)
Supplement: Translation

# Translation: Práticas de nutricionistas em unidades de diálise no Brasil: avaliação e intervenção nutricionais

**DOI:** 10.1590/2175-8239-JBN-2024-0112pt

**Authors:** Viviane de Oliveira Leal, Denise Mafra

**Affiliations:** 1 Universidade do Estado do Rio de Janeiro, Hospital Universitário Pedro Ernesto, Divisão de Nutrição, Rio de Janeiro, RJ, Brasil.; 2 Centro de Nefrologia Mageense, Magé, RJ, Brasil.; 3 Universidade Federal Fluminense, Programa de Pós-Graduação em Ciências Médicas, Niterói, RJ, Brasil.; 4 Universidade Federal do Rio de Janeiro, Programa de Pós-Graduação em Ciências Biológicas, Fisiologia, Rio de Janeiro, RJ, Brasil.

A Nutrição desempenha papel crucial na prevenção e no controle de distúrbios metabólicos e nutricionais de pacientes com doença renal crônica (DRC) em diálise. Entretanto, conforme relatado no artigo de Nerbass et al.^1^, “Práticas de nutricionistas em unidades de diálise no Brasil: avaliação e intervenção nutricionais”, o uso de ferramentas para avaliação do estado nutricional, bem como as estratégias de intervenção em casos de risco nutricional/desnutrição, ainda é heterogêneo.

Em 2020, a National Kidney Foundation, no documento *Kidney Disease Outcomes Quality Initative* (NKF/KDOQI), sugeriu diretrizes específicas para o acompanhamento nutricional de pacientes com DRC^2^. Portanto, esse *guideline* pode ser considerado o instrumento fundamental para nortear práticas relacionadas ao diagnóstico em nutrição, além de subsidiar condutas nutricionais pertinentes aos pacientes em diálise.

A triagem de risco nutricional, comumente utilizada no âmbito hospitalar, é considerada a etapa inicial, rápida e fácil que permite identificar precocemente pacientes em risco nutricional. Além da triagem, a avaliação nutricional também deve ser realizada de forma rotineira numa clínica de diálise. Uma vez identificado o déficit nutricional, o paciente deve receber maior atenção e tratamento da equipe da clínica e, obviamente, do nutricionista, que deve fazer intervenções adequadas. Assim, a avaliação nutricional de rotina e a interpretação dos dados permitem identificar as causas e a gravidade dos distúrbios relacionados à nutrição, além de nortear a terapia nutricional mais assertiva^3^.

Dessa forma, embora a triagem nutricional possa ser confundida como “mais um” instrumento a ser aplicado, ela é importante para sistematizar a necessidade de avaliação nutricional. Outro ponto interessante é que a triagem nutricional pode ser aplicada por outros profissionais de saúde, pelo próprio paciente ou sua rede de apoio. De acordo com o NKF/KDOQI (2020), a triagem de risco nutricional deve ser realizada semestralmente, e como não há indicação de um instrumento preferencial, o simples instrumento de triagem de desnutrição (do inglês, *malnutrition screening tool,* mais conhecido por sua sigla MST) pode ser utilizado. O MST inclui dados rapidamente obtidos através das fichas individuais de diálise e em anamnese com paciente (se houve perda recente de peso, de quantos quilos e se há redução de apetite).

Já a avaliação nutricional deve contemplar dados relacionados à ingestão alimentar, apetite, dados antropométricos (peso corporal, dobras cutâneas, índice de massa corporal), dados bioquímicos e exame físico^2^. Por não existir um indicador amplo e único para diagnóstico nutricional dos pacientes com DRC em diálise, os métodos compostos são ferramentas importantes na prática clínica.

A Avaliação Subjetiva Global (ASG), que engloba praticamente todas as nuances da avaliação nutricional recomendadas pelo NKF/KDOQI (2020), constitui-se o instrumento de avaliação nutricional utilizado por mais de 80% de nutricionistas no Brasil^1^. Por ter sido validada como preditora de mortalidade em pacientes com DRC, a ASG de 7 pontos é especialmente recomendada para avaliação do estado nutricional de pacientes em diálise^2^. Especificamente para pacientes em hemodiálise (HD), o *Malnutrition Inflammation Score* (MIS) também pode ser utilizado e, embora seja mais objetivo e completo que a ASG, por compreender também parâmetros bioquímicos (albumina e capacidade total de ligação do ferro) e índice de massa corporal, é utilizado por apenas 49% dos nutricionistas^1^. No entanto, vale ressaltar que o diagnóstico nutricional segundo essas ferramentas (ASG e MIS) deve, idealmente, ser complementado com parâmetros antropométricos, bioquímicos e de ingestão alimentar adicionais^4^.

A periodicidade da avaliação nutricional também é sugerida pelo NKF/KDOQI (2020)^2^: na admissão em diálise (ainda nos primeiros 90 dias de tratamento); quando indicada pela triagem nutricional; anualmente; ou segundo julgamento clinico. Na pesquisa realizada por Nerbass *et al*.^1^, a ASG foi a ferramenta que obteve maior percentual de uso com periodicidade definida (53% no total, 36% a cada três a seis meses, 9% anualmente e 8% mensalmente), provavelmente por sua facilidade de execução e interpretação.

O volume de trabalho do nutricionista é uma variável fundamental para determinar a sistematização dos cuidados aos pacientes (instrumentos utilizados e sua periodicidade). Entretanto, a diferença na variedade do uso de ferramentas utilizadas para avaliação do estado nutricional, independentemente de sua periodicidade, é pequena quando os nutricionistas são divididos segundo o número de pacientes atendidos por hora mensal de trabalho (usando o valor de corte de 1,6). Assim, é complexo afirmar se seria mais adequado usar menos ferramentas em intervalo mais curto ou mais ferramentas com periodicidade de aplicação maior.

Entretanto, é fato que 1,6 paciente por hora de trabalho do nutricionista está bem acima do sugerido pelo Conselho Federal de Nutricionistas, que estabelece 1 nutricionista para cada 50 pacientes em carga horária mensal de 30 horas semanais (CFN, 2018)^5^, o que corresponderia ao ideal de 0,3 paciente por hora de trabalho. Portanto, é interessante que cada unidade de diálise estabeleça suas prioridades e potencialidades em rotina técnica própria, considerando a gestão de tempo e os recursos disponíveis de cada serviço com o objetivo de priorizar não apenas o diagnóstico, mas também a continuidade do cuidado nutricional.

A intervenção nutricional nos casos de desnutrição, observada no estudo de Nerbass *et al.* ^1^, segue o preconizado pelas diretrizes internacionais, que determinam o aconselhamento nutricional como estratégia inicial de intervenção^2^. Assim, o maior fracionamento e/ou porcionamento alimentar, além da inclusão de alimentos de alta densidade energética e/ou proteica, é indicado. Para monitorar a adesão, bem como prover ajustes pertinentes, o aumento da periodicidade de atendimento é necessário e, de fato, descrito pela maioria dos nutricionistas.

Em situações em que ainda houver lacuna entre a ingestão real e as necessidades-alvo/objetivos do tratamento nutricional, a suplementação nutricional oral (SNO) deve ser considerada. Para isso, alguns detalhes são importantes^2,6^:

- O tempo de seguimento para SNO deve ser de, no mínimo, 3 meses;
- A SNO deve ser prescrita 2 a 3 vezes ao dia e, para que não haja comprometimento da ingestão alimentar habitual (devido a maior saciedade), a suplementação deve ser realizada 1 hora após as principais refeições. Outra opção seria o uso como última refeição do dia (ceia), como forma de atenuar o jejum noturno ou mesmo durante o procedimento de HD para amenizar/reverter o catabolismo. Nesse caso, possíveis intercorrências (como sintomas gastrintestinais e hipotensão pós-prandial) devem ser contornadas com monitoramento rigoroso;
- A prescrição da SNO deve ser feita segundo a preferência do paciente, considerando diferentes apresentações possíveis (barras, pó, preparados prontos);
- Nos casos em que não houver necessidade de restrição hídrica e/ou de eletrólitos, podem ser utilizadas fórmulas de composição-padrão, sempre com monitoramento intensivo.

Esses detalhes são cruciais para a escolha da SNO a ser utilizada, já que haverá necessidade de uso por período considerável, o que determina maior custo. Além disso, as características sensoriais do suplemento/módulo escolhido devem ser agradáveis o suficiente para favorecer a adesão prolongada.

No Brasil, apenas 17% das unidades de diálise fornecem suplementação (para todos ou apenas a alguns dos pacientes com indicação). Esse percentual é variável entre as regiões do país^1^. Desse modo, políticas públicas específicas para doação de suplementos ou viabilidade de recursos financeiros adicionais para pacientes em diálise com desnutrição e em situação de vulnerabilidade social poderiam ser articuladas nacionalmente.

Como na maioria das vezes os próprios pacientes precisam arcar com os custos da SNO, a utilização de módulos de nutrientes (indicados por 63% dos nutricionistas) ou uso de fórmulas industrializadas precisam ser consideradas de acordo com as necessidades clínicas e bioquímicas individuais, bem como disponibilidade financeira. Surpreendentemente, o percentual de indicação de fórmulas-padrão e específicas para DRC em diálise não foi diferente (cerca de 78%), embora o custo das fórmulas específicas seja consideravelmente maior.

Embora possa parecer mais seguro, as fórmulas industrializadas específicas para pacientes em diálise não são isentas de potássio e fósforo e, portanto, a escolha deve ser cuidadosa. As fórmulas-padrão também apresentam conteúdo muito variável de eletrólitos. Contudo, a possibilidade de uso de versões sem sabor pode tornar mais versátil a intervenção nutricional, já que há possibilidade de uso em diferentes tipos de preparações (por exemplo, leite em vitaminas e mingaus, em massas de panquecas ou preparações salgadas, como purês e sopas).

Considerando todas essas possibilidades para avaliação do estado nutricional e principalmente para intervenções nutricionais assertivas, fica ainda mais evidente a importância do nutricionista no acompanhamento dos pacientes em diálise, já que o cuidado nutricional adequado e individualizado é pilar fundamental do tratamento de pacientes com DRC em diálise.

Com relação à pesquisa realizada por Nerbass *et al*.^1^, ressalta-se a necessidade de maior engajamento por parte dos nutricionistas das clínicas nesse tipo de pesquisa, pois apenas 24% responderam ao questionário. Um questionário eletrônico divulgado em mídias sociais e aplicativos de mensagens é apropriado para alcançar grande número de profissionais. No entanto, pode introduzir um viés de amostragem, como discutido pelas autoras.

Por fim, o estudo aborda um tema relevante e necessário sobre as práticas nutricionais em unidades de diálise no Brasil, fornecendo material para que estudos futuros abordem de forma mais aprofundada a variabilidade das práticas nutricionais, bem como forneçam análises regionais mais detalhadas.
